# The Future of Bart's

**Published:** 1904-01-23

**Authors:** Trevor Lawrence

**Affiliations:** Treasurer


### EDITOR'S LETTER-BOX.

**[Our Correspondents are reminded that prolixity is a great bar to publication, and that brevity of style and conciseness of statement greatly facilitate early insertion.]**

### THE FUTURE OF BART'S.

*To the Editor of* "THE HOSPITAL."

SIR,—As the future of St. Bartholomew's Hospital is of great importance to the City of London, to the sick poor, and to the cause of medical education, and as the date of our appeal is now near at hand, I trust you will allow me to place before your readers the present position of affairs, which seems to me, if I may judge by the statements I have seen in the Press, to be but very imperfectly understood.

First, let me say a few words regarding the proposed removal of the hospital from its present site. This question was gone into very thoroughly by the Mansion House Committee, who devoted four sittings to hearing evidence on it. The result was that the Committee decided, with one dissentient, "that it was impossible, in the public interest, to entertain the idea of removing St. Bartholomew's Hospital from its present site." The one dissentient was a member who was known to have settled convictions regarding the removal of hospitals.

The Mansion House Committee next discussed the question of the adequacy of the present site, 6½ acres in extent, to provide room for the buildings of a "hospital with every modern appliance." Although they decided that the site was adequate, it became evident, during the autumn, when the disposition of the many necessary buildings came to be discussed, that the weight of public and professional opinion was against their conclusion. This led to conferences between the Governors and the Medical Staff of the hospital. The result was that the Governors, in compliance with the representations of their staff, agreed to endeavour to find a site for the Nurses' Home, which would have to accommodate a female staff numbering 320, outside the hospital precincts—the remaining area of Christ's Hospital site having meanwhile been bought by the Post Office.

During the sittings of the Mansion House Committee and the whole of the latter part of last year the hospital architect, Mr. I'Anson, who had temporarily associated with him Mr. Rowland Plumbe (one of the architects of the London Hospital), has been engaged in preparing plans for the distribution of the new buildings contemplated. But when the Governors, in concert with the medical staff, proposed to put the nurses' home outside the hospital area, these plans necessarily required revision. They have been carefully revised, and block plans, showing how the hospital buildings of the future may be distributed in more ways than one, have been prepared and submitted to the Medical Council. It has been urged that until a cut and dried plan for the complete rebuilding of the hospital is laid before the public it is undesirable to make any appeal for funds. This is not the opinion of a great majority of the Governors and of the medical staff. Meanwhile the number of blocks, the purposes for which they are required, and the estimated cost of each, and of the nurses' home, have long been known, and can be put distinctly before the meeting of the 26th instant. The public will be content, I believe, to leave the Governors, in consultation with their professional advisers and their scientific staff, to deal with all questions of detail, for which purpose it is proposed to appoint a Special Building Committee of Governors and of members of the medical staff.

It is necessary in this letter to refer to a few general considerations. St. Bartholomew's is the only general hospital within the City limits, and has carried on its work on the same site for nearly 800 years. The contemplated removal of King's College Hospital will throw a permanently increased burden upon St. Bartholomew's, in the same way as the London Hospital has found itself subject to severe pressure temporarily whenever a wing of this hospital has had to be closed. The patients treated here during the last fifty years have exceeded 7,000,000—surely a striking argument against the removal of the hospital.

With regard to the financial position of the hospital, into which the Mansion House Committee carefully examined, they report that the "recent purchase of land from Christ's Hospital will entail a charge of over £9,000 per annum on its present revenue, leaving a deficit of £7,000 per annum on the ordinary expenditure."

Were the suggestion adopted that our appeal should be again deferred, it must be pointed out that this would involve the waste of a second year's interest on the purchase money of the land referred to.

St. Bartholomew's has not asked the public for help for more than 150 years. Repeated and emphatic representations from the hospital staff, representations believed by the Governors to be well-grounded and not over-coloured, have set out a long list of costly new buildings which are ugently required if the hospital and its medical school are to maintain their world-wide renown.

The funds for these buildings, and for rebuilding the wards, cannot be supplied from the resources of the hospital itself without grievously curtailing its work and the benefits it confers on the sick poor. It would probably be necessary to close several of the wards, although the beds are not now one too many. How, then, can the funds required be obtained except by appealing to the public and to the never-failing and inexhaustible generosity of the citizens of the greatest city in the world?

In conclusion, let me quote the final paragraph of the Mansion House Committee's report: "The Committee do not think that they would be justified in concluding their functions without placing upon record their opinion that from the evidence brought before them the administration of the hospital has been conducted by the Governors in a wise and enlightened spirit, with a due regard to economy, and in the best interests of the patients."—Yours, etc.,

Trevor Lawrence, Treasurer.

